# Different profiles among older adults with HIV according to their chronological age and the year of HIV diagnosis: The FUNCFRAIL cohort study (GeSIDA 9817)

**DOI:** 10.1371/journal.pone.0266191

**Published:** 2022-03-30

**Authors:** Fátima Brañas, Mª José Galindo, Miguel Torralba, Antonio Antela, Jorge Vergas, Margarita Ramírez, Pablo Ryan, Fernando Dronda, Carmen Busca, Isabel Machuca, Mª Jesús Bustinduy, Alfonso Cabello, Matilde Sánchez-Conde, on behalf of the FUNCFRAIL study group

**Affiliations:** 1 Geriatrics Department, Hospital Universitario Infanta Leonor, Madrid, Spain; 2 Fundación para la Investigación e Innovación Biomédica H.U Infanta Leonor y H.U. Sureste, Universidad Complutense, Madrid, Spain; 3 Internal Medicine/ Infectious Diseases Department, Hospital Universitario Clínico de Valencia, Valencia, Spain; 4 Internal Medicine Department, Hospital Universitario de Guadalajara, Universidad de Alcalá, Madrid, Spain; 5 Infectious Diseases Unit, Hospital Clínico Universitario de Santiago de Compostela, Universidad de Santiago de Compostela, Santiago de Compostela, Spain; 6 Internal Medicine/ Infectious Diseases Department, Hospital Universitario Clínico San Carlos, Madrid, Spain; 7 Infectious Diseases Unit, Hospital General Universitario Gregorio Marañón, Madrid, Spain; 8 HIV Clinic, Hospital Universitario Infanta Leonor, Madrid, Spain; 9 Infectious Diseases Department, Hospital Universitario Ramón y Cajal, IRYCIS, Madrid, Spain; 10 HIV Unit/Internal Medicine Department, Hospital Universitario La Paz, IdiPAZ, Madrid, Spain; 11 Infectious Diseases Department, Hospital Universitario Reina Sofía, Córdoba, Spain; 12 Infectious Diseases Department, Hospital de Donostia, San Sebastián, Spain; 13 Infectious Diseases Department, Fundación Jiménez Díaz, Madrid, Spain; Brighton and Sussex Medical School, UNITED KINGDOM

## Abstract

**Background:**

People in their fifties with HIV are considered older adults, but they appear not to be a homogeneous group.

**Objective:**

To evaluate the differences among older adults with HIV according to their chronological age and the year of HIV diagnosis.

**Methods:**

Cross-sectional study of the FUNCFRAIL cohort. Patients 50 or over with HIV were included and were stratified by both chronological age and the year of HIV diagnosis: before 1996 (long-term HIV survivors [LTHS]) and after 1996. We recorded sociodemographic data, HIV-related factors, comorbidities, frailty, physical function, other geriatric syndromes, and quality of life (QOL).

**Results:**

We evaluated 801 patients. Of these, 24.7% were women, 47.0% were LTHS, and 14.7% were 65 or over. Of the 65 or over patients, 73% were diagnosed after 1996. Higher rates of comorbidities among LTHS were found, being the more prevalent: COPD, history of cancer, osteoarthritis, depression, and other psychiatric disorders while the more prevalent among the 65 or over patients were: hypertension, diabetes, dyslipidemia, cancer, and osteoarthritis. LTHS showed a significantly worse QOL. There were no differences by the year of HIV diagnosis regarding frailty and functional impairment (SPPB <10) but they were more than twice as prevalent in the 65 or over patients compared to the other chronological age groups.

**Conclusions:**

A LTHS and a 65 or over person are both “older adults with HIV,” but their characteristics and requirements differ markedly. It is mandatory to design specific approaches focused on the real needs of the different profiles.

## Introduction

People in their fifties with HIV are considered older adults because at this age, the immunological recovery is lower and slower after antiretroviral treatment (ART) than in younger people [1] and age-related comorbidities, frailty, and geriatric syndromes become more prevalent [2–4]. Half of the people with HIV nowadays are older adults [5] and predictions point to an exponential increase of this proportion [6]. Characteristics of aging are not homogeneous. At the same chronological age, the health status can be absolutely different [7], considering the World Health Organization’s (WHO) definition of health, “the process of developing and maintaining the functional ability that enables wellbeing in older age”, which takes into account (beyond comorbidities) the physical function status, the presence of frailty, and the quality of life (QOL) [8]. Frailty and functional impairment have proven to be better predictors of survival than comorbidity per se, specifically of disability-free years of life in the general population [9–11]. Physical frailty is a clinical syndrome defined by five criteria—shrinking, weakness, poor endurance and energy, slowness, and low physical activity level—in which the whole is more than the sum of the parts in terms of predicting adverse health outcomes [12]. Frailty is prevalent in the older adults with HIV, is associated with mortality and is potentially reversible [13–15].Frailty is starting to be proposed as a clinical marker of biological age [7].

The growing number of older adults with HIV is due to ART’s success at improving survival for people with HIV and to the increase in the number of new cases diagnosed in this age group [5]. All these people are now considered “older adults with HIV” despite appearing to have different characteristics and needs. Consequently, it is crucial to know if there are differences between older adults with HIV aged 50 years or over, according to their chronological age and the year of HIV diagnosis in terms of HIV variables, comorbidity, and social factors, but also in terms of frailty, physical function, other geriatric syndromes, and QOL. This is the main purpose of our study.

## Materials and methods

### Study design and patient population

We performed a cross-sectional study of the FUNCFRAIL cohort (Spanish cohort) to study frailty and physical function in older adults with HIV. Recruitment took place between May 2018 and November 2019. The inclusion criteria were confirmed HIV infection, age ≥50 years at the time of recruitment, and regular follow-up at the HIV clinic. We recruited 801 patients from eleven hospitals in Spain who were then included in a random way, including patients who agreed to participate and signed the written informed consent. The study was approved by the Ethics Committee of Hospital Universitario Ramón y Cajal, Hospital Universitario Gregorio Marañón (approval was extended for the Hospital Universitario Infanta Leonor), Hospital Universitario Clínico San Carlos, Hospital Universitario de Guadalajara, Hospital Universitario La Paz, Hospital Universitario Reina Sofía, Fundación Jiménez Díaz and the Regional Ethics Committee of Galicia, Euskadi and Comunidad Valenciana. Patients were stratified into three groups according to their chronological age at the time of recruitment: 50–54 years (younger), 55–64 years (intermediate age group), and 65 years or over (65 or over). In addition, the whole sample was stratified into two groups according to the year of HIV diagnosis: 1996 or before (long-term HIV survivors [LTHS]) and after 1996 (HAART-era group).

### Data collection

We recorded sociodemographic data, HIV infection-related data, medications (polypharmacy defined as taking ≥ 5 co-medications other than ART), and comorbidity due to self-reported, physician-diagnosed chronic conditions: hypertension, type 2 diabetes, dyslipidemia, coronary heart disease, stroke, COPD, chronic kidney disease, cancer (< 5 years from the diagnosis), history of cancer (≥ 5 years from the diagnosis; not active disease), depression, psychiatric disease, and osteoarticular disease.

A comprehensive geriatric assessment was performed. Frailty was defined according to Fried’s criteria [10]; namely, shrinking (unintentional weight loss of ≥ 4.5 kg or ≥ 5% of body weight during the previous year), weakness (grip strength adjusted for gender and body mass index), poor endurance and energy (self-reported exhaustion identified by two questions from the Center for Epidemiologic Studies Depression scale), slowness (based on time to walk 4.6 meters, adjusting for gender and standing height), and low physical activity level (< 383 kcal/week in men and < 270 kcal/week in women using the Minnesota Leisure Time Activity Questionnaire). Patients were considered frail if they met at least three of the five criteria, prefrail when they met one or two criteria, and robust when they met no criteria. Physical function was assessed with the objective measures of strength, gait speed, and balance using the Short Physical Performance Battery (SPPB) [16]. Falls were evaluated by self-reporting in response to the question: “Have you fallen in the past year?” Cognitive impairment was evaluated using the Montreal Cognitive Assessment test (MOCA) [17], and depression was tested using the Short-Form Geriatric Depression Scale (SF-GDS) [18]. Each patient performed a QOL self-assessment by stratifying their QOL into one of the following categories: very good, good, fair, or poor. We also evaluated the QOL by asking the direct question of the SF-GDS: “Are you satisfied with your life?” with the dichotomous answer of yes or no [18]. We also evaluated QOL by the presence of pain because pain is considered a marker of QOL.

### Statistical analysis

We used descriptive statistics to examine the participants’ characteristics, which are expressed as frequencies (percent) of categorical variables, mean (SD) of normally distributed continuous variables, and median (P25-P75) of continuous variables with a skewed distribution. Continuous variables were compared using the *t* test for independent variables. The Mann-Whitney test was used for variables with a non-normal distribution or when the group size was small. The association between qualitative variables was assessed using the chi-square test or Fisher’s exact test when a group was very small. We compared the four resulting groups from the combination of the chronological age and the year of HIV diagnosis with one another, resulting in a total of six comparisons. For that purpose, comparing three or more groups simultaneously, the Bonferroni adjustment was applied to correct for a possible increase in type 1 errors (false positives). Statistical analysis was performed with IBM SPSS Statistics for Windows software (Version 25.0. Armonk, NY: IBM Corp). All statistical tests were two-sided, and *P* values < .05 were considered statistically significant. The *P* set by Bonferroni was .05/6, as we made six comparisons, so *P* values < .0083 were considered statistically significant in this analysis. There was no imputation in the missing values; we worked with observed data.

## Results

We evaluated 801 older adults with HIV. One in four were women (195 [24.7%]); the median age was 56.61 (53.7–61.14) years, 14.7% were 65 or over, and 47.02% were LTHS.

### Sociodemographic characteristics, HIV-related factors, and comorbidity

See Table 1 for more details.

**Table 1:** Sociodemographic characteristics, HIV-related factors, comorbidity, and medications.

		Total	Chronological Age				Year of HIV diagnosis		
			50–54	55–64	≥ 65	p	≤ 1996	>1996	p
Sociodemographic characteristics	Patients. N (%)	801	290 (36.2)	393 (49)	118 (14.7)		372 (47.0)	419 (52.9)	
	Women. N (%)	195 (24.7)	83 (28.6)	89 (22.6)	24 (20.3)	0.1	115 (30.9)	80 (19.1)	<0.001
	Age. Median (p25-75)	56.6 (53.7–61.1))	52.7 (51.5–53.9)	58 (56.3–60.6)	69 (66.5–73)	<0.001	56.2 (53.9–60)	57.1 (53.4–63.5)	0.03
	Chronological age group. Years. N (%)								
	50–54	290 (36.2)	-	-	-		131 (35.2)	153 (36.5)	<0.001
	55–64	393 (49)					209 (56.2)	180 (43)	
	≥ 65	118 (14.7)					32 (8.6)	86 (20.5)	
	Education. N (%)								
	Illiterate	6 (0.8)	4 (1.5)	2 (0.6)	-		3 (0.9)	3 (0.8)	
	Primary school	322 (44.4)	123 (45.8)	148 (42.8)	51 (46.8)	0.06	165 (49.3)	154 (40)	0.002
	High school	190 (26.2)	66 (24.5)	96 (27.7)	28 (25.7)		93 (27.8)	96 (24.9)	
	Short-cicle, diploma	88 (12.1)	34 (12.6)	42 (12.1)	12 (11)		40 (11.9)	48 (12.5)	
	University	118 (16.3)	42 (15.6)	58 (16.8)	18 (16.5)		34 (10.1)	84 (21.8)	<0.001
	Living. N (%)								
	Alone	299 (37.3)	94 (32.5)	148 (37.9))	58 (49.2)	0.007	114 (30.7)	185 (44.3)	<0.001
	With partner	332 (41.4)	132 (45.7)	162 (41.5)	42 (35.6)		166 (44.2)	166 (39.7)	
	With children/caregiver	158 (19.7)	63 (21.8)	78 (20)	18 (15.3)		91 (24.5)	67 (16)	0.003
	Nursing home	2 (0.2)	0	2 (0.5)	0		2 (0.5)	-	
HIV-related factors	Age at HIV diagnosis. Median (p25-75)	36.2 (29.5–46.3))	31.3 (25–40.1)	35.3 (29–43.6)	53.5 (45.8–59)	<0.001	28.1 (24.6–32.3)	45.3 (38.6–51.9)	<0.001
	Years with known HIV infection. Median (p25-75))	21.5 (13.6–27.6))	21.1 (11.6–27.4)	23.2 (16.1–28.8)	17.2 (11.1–22.8)	<0.001	28.05 (25.3–31.2)	14.1 (7.7–19)	<0.001
	Year of HIV diagnosis. N (%)								
	≤1996 >	372 (47.02)	131 (46.1)	209 (53.7)	32 (27.1) 86	<0.001	-	-	
	1996	419 (52.9)	153 (53.9)	180 (46.3)	(72.9)				
	Risk practice for HIV infection. N (%)								
	IDU	286 (35.9)	109 (37.8)	171 (43.8)	6 (5.1)		225 (60.6)	59 (14.1)	
	MSM	242 (30.5)	88 (30.6)	98 (25.1)	57 (48.3)	<0.001	60 (16.2)	183 (43.9)	<0.001
	Heterosexual	202 (25.3)	73 (25.3)	90 (23.1)	39 (33.1)		66 (17.8)	135 (32.4)	
	NA	65 (8.1)	18 (6.2)	31 (7.2)	16 (13.5)		20 (5.4)	40 (9.4)	
	B or C CDC stage. N (%)	402 (50.1)	149 (52.1)	203 (53.3)	53 (47.3)	0.1	222 (61.3)	180 (44)	0.03
	Nadir CD4+ T-cell. Median (p25-75)	195 (87–317)	197 (68–340)	195 (100–334)	184 (78–270)	0.008	182 (88–290)	207 (86–344)	0.09
	Undetectable HIV RNA. N (%)	724 (90.3)	256 (89.5)	357 (92.5)	111 (94.1)	0.2	340 (91.9)	377 (91.7)	1
	Current CD4+ T-cell Median (p25-75)	677 (480–903)	726 (514–977)	677 (470–884)	589 (462–780)	0.001	688.5 (487–946)	669.9 (471–879.5)	0.1
	CD4/CD8 ratio. Median (p25-75)	0.79 (0.5–1.1)	0.82 (0.5–1.1)	0.76 (0.5–1.1)	0.74 (0.5-1-0)	0.8	0.76 (0.5–1.0)	0.80 (0.5–0.8)	0.3
Comorbidity and medications	Alcohol intake. N (%)	81 (10.3)	29 (10.1)	45 (11.7)	7 (6.1)	0.2	40 (11)	38 (9.2)	0.4
	Current smoker. N (%)	356 (45.2)	149 (52.1)	188 (48.8)	19 (16.4)	<0.001	195 (53.6)	154 (37.2)	<0.001
	Comorbidities. Mean (SD)	2.2 (1.7)	1.9 (1.7)	2.3 (1.7)	2.6 (1.6)	<0.001	2.6 (1.8)	1.8 (1.6)	0.002
	Comorbidities. N (%)								
	≤ 2.	493 (61.5)	195 (67.2)	234 (59.5)	64 (54.2)	0.01	193 (51.9)	292 (69.7)	
	3–4	217 (27)	65 (22.4)	112 (28.5)	40 (33.9)	0.01	121 (32.5)	94 (22.4)	<0.001
	≥ 5	91 (11.3)	30 (10.3)	47(12)	14 (11.9)	0.7	58 (15.6)	33 (7.9)	
	Polypharmacy. N (%)	209 (26.2)	73 (25.3)	95 (24.4)	41 (34.7)	0.03	117 (31.5)	90 (21.6)	<0.001
	Nº of medications^†^. Mean (SD)	3 (4)	2.8 (3.0)	3.2 (2.6)	4.4 (4)	0.001	3.6 (3.0)	2.9 (3.1)	<0.001
	Specific medications. N (%)								
	Neuroleptics	90 (11.2)	33 (11.4)	50 (12.7)	7 (5.9)	0.04	62 (16.7)	28 (6.7)	<0.001
	Benzodiazepines	166 (20.7)	68 (22.8)	82 (20.9)	18 (15.3)	0.2	102 (27.4)	63 (15)	<0.001
	Hypnotics	43 (5.3)	12 (4.1)	28 (7.1)	3 (2.5)	0.07	32 (8.6)	11 (2.6)	<0.001
	First step analgesics	117 (14.6)	42 (14.5)	64 (16.3)	11 (9.3)	0.1	59 (15.9)	56 (13.4)	0.3
	Opioids	32 (4)	14 (4.8)	12 (3.1)	6 (5.1)	0.4	16 (4.3)	16 (3.8)	0.8

IDU: injection drugs user. MSM: men who have sex with men. NA: not available. CDC: Centers for Control Diseases. Polypharmacy: ≥ 5 medications.

^†^Number of medications excluding ART.

#### Stratified by chronological age

**Fig 1:**
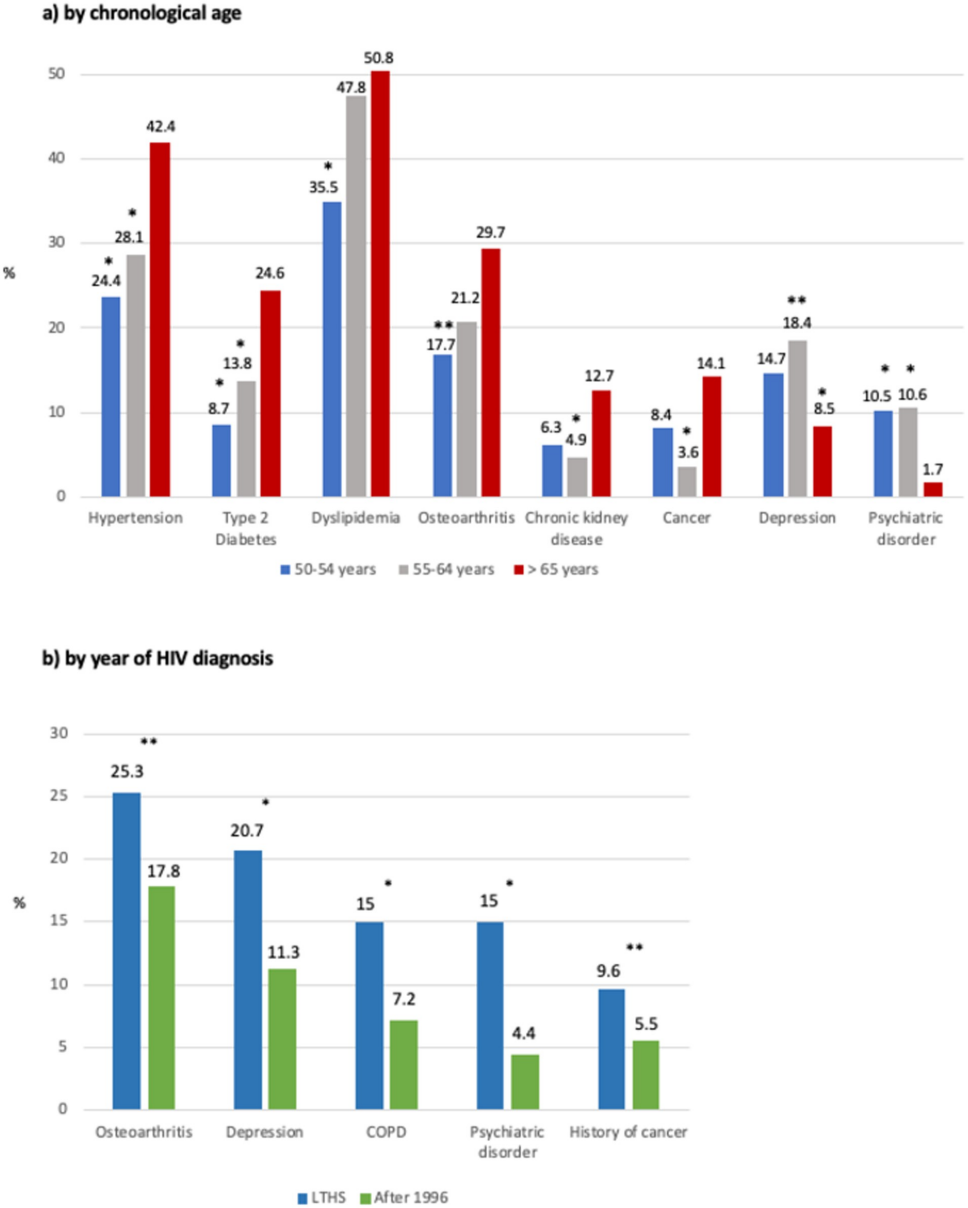
Comorbidities stratified both by chronological age and year of HIV diagnosis. * Significant difference with respect to 65 or over group (p < 0.005) ** Significant difference with respect to 65 or over group (p < 0.01).

Patients 65 or over were 20 years older at HIV diagnosis than the youngest patient and had been living with known HIV for fewer years at recruitment. Three in four of patients 65 or over were diagnosed after 1996. No significant differences were found between the three groups in terms of virological response to ART, but differences were found regarding immune status as measured by the CD4 T-cell nadir and the current CD4 T-cell count, which were both lower among patients 65 or over. Regarding comorbidities, the proportion of patients with no associated comorbidity decreased significantly with age, and the proportion of patients with 3–4 comorbidities was significantly higher among patients 65 or over compared to the youngest (33.9% vs 22.4% [p = 0.01]). Polypharmacy was significantly higher among patients 65 or over compared to the other two age groups. There were differences between age groups concerning some comorbidities represented in Fig 1a. No differences were found as to the history of cancer, heart attack, stroke, or COPD.

#### Stratified by the year of HIV diagnosis

Women represented 30.9% of the LTHS, a proportion significantly higher than in the HAART-era group with 19.1% (p <0.001). 56.2% of LTHS were 55–64 years at the time of recruitment; only 8.6% were 65 or over, while 20.5% of the HAART-era group were in the oldest group. Regarding the risk practice for HIV infection, 60.6% of the LTHS were people who used injectable drugs while 76.3% of the HAART-era group were sexual contact. No significant differences were found for immunovirological status. The mean of comorbidities was significantly higher in the LTHS, and the proportion of patients with five or more comorbidities was twice as high among LTHS as in the HAART-era group. Polypharmacy was significantly more prevalent in the LTHS, as was the use of neuroleptics, benzodiazepines, and hypnotics. There were differences between LTHS and the HAART-era group regarding some comorbidities, as shown in Fig 1b. No differences were found in relation to hypertension, diabetes, dyslipidemia, heart attack, stroke, chronic kidney disease, or cancer.

### Frailty, physical function, other geriatric syndromes, and quality of life

#### Stratified by chronological age

**Fig 2:**
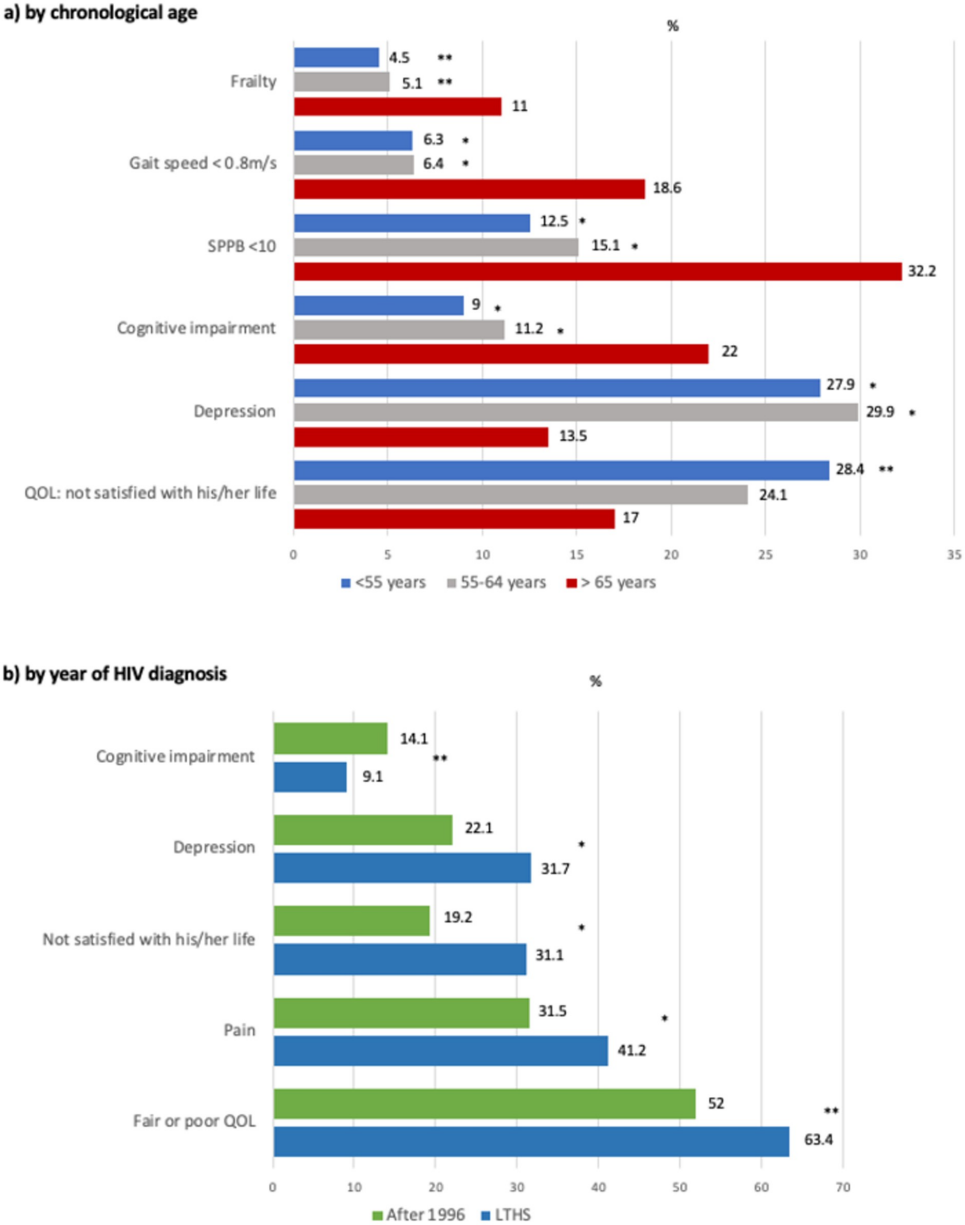
Frailty, physical function, other geriatric syndromes, and quality of life stratified both by chronological age and year of HIV diagnosis. Gait speed < 0.8m/s indicates poor health and functional status and suggests worse than average life expectancy. SPPB: Short Physical Performance Battery. SPPB <10 indicates functional impairment. QOL: quality of life. * Significant difference with respect to 65 or over group (p < 0.005) ** Significant difference with respect to 65 or over group (p < 0.01).

Frailty was more than twice as prevalent in patients 65 or over compared to the other chronological age groups, as shown in Fig 2a. The proportion of patients 65 or over with an SPPB score under 10, which indicates functional impairment, was double that of the other age groups, and the proportion of patients 65 or over with a gait speed of less than 0.8m/s was three times higher than in the younger patients. For patients 65 or over, 18.6% had a gait speed over 1.2m/s. No differences were found among the three chronological age groups (youngest, intermediate age, and 65 or over group) in the prevalence of prefrailty (54.9% vs. 49.7% vs. 55.9%) or falls (13.5% vs.17.9% vs.13.6%). Cognitive impairment, defined as a MOCA test score ≤ 20 points, was twice as prevalent among patients 65 or over than in the other groups, while depressive symptoms, determined by SF-GDS ≥ 6 points, were half that of the younger patients. There were no differences in QOL stratification, but differences were found between patients 65 or over and the youngest of the older adults when asked, “Are you satisfied with your life?” See Fig 2a for more details.

#### Stratified by the year of HIV diagnosis

No significant differences were found between LTHS and the HAART-era group in terms of frailty (4.8% vs. 6.5%), prefrailty (54.8% vs. 49.9%), or falls (17.7% vs. 13.9%). There also were no differences found regarding physical function as measured by SPPB (SPPB < 10 [19.1% vs. 14.7%]) or gait speed (gait speed < 0.8m/s [5.9% vs. 9.4%,]); gait speed > 1.2m/s (37.1% vs. 42.9%), except for the proportion of patients with gait speed scores between 0.8m/s and 1.2m/s (57% vs. 47.7%, [p = 0.01]). Cognitive impairment was significantly lower among LTHS, while depressive symptoms, determined by SF-GDS ≥ 6 points, were significantly higher than in the HAART-era group patients. QOL was significantly worse among LTHS compared to the HAART-era group patients, as shown in Fig 2b.

### Comparison by groups combining chronological age and year of HIV diagnosis

We compared the four resulting groups from the combination of the chronological age and the year of HIV diagnosis with one another, resulting in a total of six comparisons. Comparison 1 was between patients 65 or over diagnosed in 1996 or before and those 65 or over diagnosed after 1996. Comparison 2 was between patients younger than 65 years diagnosed in 1996 or before and those younger than 65 years diagnosed after 1996. Comparison 3 was between patients 65 or over diagnosed in 1996 or before and patients younger than 65 years diagnosed in 1996 or before. Comparison 4 was between patients 65 or over diagnosed after 1996 and patients younger than 65 years diagnosed after 1996. Comparison 5 was between patients 65 or over diagnosed in 1996 or before and those younger than 65 years diagnosed after 1996. Comparison 6 was between patients younger than 65 years diagnosed in 1996 or before and those 65 or over diagnosed after 1996. No differences were found among patients 65 or over regardless of the year of HIV diagnosis (Comparison 1), except for the age at HIV diagnosis (pre-1996 43.3 [5.2] vs post-1996 56.9 [7.5], p = 0.0001) and the years lived with known HIV (pre-1996 26.5 [3] vs post-1996 13.4 [6.1], p = 0.0001). The differences between patients 65 or over diagnosed in 1996 or before (n = 32) compared to patients younger than 65 years diagnosed in 1996 or before (n = 340) (Comparison 3) concerned age at HIV diagnosis (43.3 [5.2] vs 27.8 [4.8]), years with known HIV infection (26.5 [3] vs 28.3 [3.5]), risk practice for HIV infection, smoking status, depression, and QOL.

There were major differences among patients younger than 65 according to the year of HIV diagnosis regarding HIV-related factors, comorbidity and medications, function, other geriatric syndromes, and QOL. When comparing patients 65 and over with the patients younger than 65, significant differences were found, especially among those diagnosed after 1996. The results of the comparison 2, 4, 5 and 6 are shown in Table 2.

**Table 2:** Comparison by groups combining chronological age and year of HIV diagnosis.

	≥ 65 post vs < 65 pre-1996			< 65 pre vs post-1996			Post-1996 < 65 vs ≥ 65			<65 post vs ≥ 65 pre-1996		
	> 1996 ≥ 65	≤ 1996 < 65	p	≤ 1996 < 65	> 1996 < 65	p	> 1996 < 65	>1996 ≥65	p	>1996 <65	≤ 1996 ≥ 65	p
Patients. N	86	340		340	333		333	86		333	32	
Women. N (%)	19 (22.1)	110 (32.4)	-	110 (32.4)	61 (18.3)	**	61 (18.3)	19 (22.1)	-	61 (18.3)	5 (15.6)	-
Living alone. N (%)	40 (46.5)	96 (28.3)	*	96 (28.3)	145 (43.7)	**	145 (43.7)	40 (46.5)	-	145 (43.7)	18 (56.3)	-
Age at HIV diagnosis. Median (p25-75)	56.6 (52–61.5)	27.5 (24.4–31)	**	27.5 (24.4–31)	42.8 (37–49)	**	42.8 (37–49)	56.6 (52–61.5)	**	42.8 (37–49)	43.5 (39.6–46.3)	-
Years with known HIV infection. Median (p25-75))	14.2 (9.8–18.3)	28.3 (25.3–31.1)	**	28.3 (25.3–31.3)	14 (7.5–19.1)	**	14 (7.5–19.1)	14.2 (9.8–18.3)	-	14 (7.5–19.1)	26.1 (23.9–28.3)	**
Risk practice for HIV infection. N (%)												
IDU	2 (2.3)	221 (65.2)	**	221 (65.2)	57 (17.2)	**	57 (17.2)	2 (2.3)	**	57 (17.2)	4 (12.5)	-
MSM	40 (46.5)	43(12.7)	**	43 (12.7)	143 (43.2)	**	143 (43.2)	40 (46.5)	-	143 (43.2)	17 (53.1)	-
Heterosexual	31 (36)	58 (17.1)	**	58 (17.1)	104 (31.4)	**	104 (31.4)	31 (36)	-	104 (31.4)	8 (25)	-
B CDC stage. N (%)	9 (10.8)	82 (24.6)	*	82 (24.6)	45 (13.8)	**	45 (13.8)	9 (10.8)		45 (13.8)	2 (6.9)	-
Nadir CD4+ T-cell. Median (p25-75)	160 (71–260)	180 (87–287)	-	180 (87–287)	229.5 (96–364)	*	229.5 (96–364)	160 (71–260)	*	229.5 (96–364)	197.5 (119.5–324)	-
Current CD4+ T-cell. Median (p25-75)	571 (441–748)	685 (487–946)	*	685 (487–946)	694 (484–919)	-	694 (484–919)	571 (441–748)	*	694 (484–919)	721.4 (517.3–947)	-
Current smoker. N (%)	12 (14.1)	188 (56.5)	**	188 (56.5)	142 (43.2)	**	142 (43.2)	12 (14.1)	**	142 (43.2)	7 (22.6)	-
Nª comorbidities. Mean (SD)	2.5 (1.6)	2.6 (1.8)	-	2.6 (1.8)	1.7 (1.5)	**	1.7 (1.5)	2.5 (1.6)	**	1.7 (1.5)	2.8 (1.8)	**
Comorbidities. N (%)												
≤ 2	47 (54.7)	176 (51.8)	-	176 (51.8)	245 (73.6)	**	245 (73.6)	47 (54.7)	-	245 (73.6)	17 (53.1)	-
3–4	28 (32.6)	109 (32.1)	-	109 (32.1)	66 (19.8)	**	66 (19.8)	28 (32.6)	*	66 (19.8)	12 (37.5)	-
≥ 5	11 (12.8)	55 (16.2)	-	55 (16.2)	22 (6.6)	**	22 (6.6)	11 (12.8)	-	22 (6.6)	3 (9.4)	-
Specific comorbidities. %												
Hypertension	33 (38.4)	100 (29.6)	-	100 (29.6)	78 (23.5)	-	78 (23.5)	33 (38.4)	-	78 (23.5)	17 (53.1)	**
Diabetes Mellitus	20 (23.3)	50 (14.8)	-	50 (14.8)	28 (8.5)	-	28 (8.5)	20 (23.3)	**	28 (8.5)	9 (28.1)	**
Cancer	15 (17.6)	21 (6.2)	*	21 (6.2)	17 (5.1)	-	17 (5.1)	15 (17.6)	**	17 (5.1)	2 (6.3)	-
Osteoarthritis	26 (30.2)	83 (25)	-	83 (25)	48 (14.6)	**	48 (14.6)	26 (30.2)	*	48 (14.6)	9 (28.1)	-
Depression*	9 (10.6)	75 (22.3)	-	75 (22.3)	38 (11.5)	**	38 (11.5)	9 (10.6)	-	38 (11.5)	1 (3.1)	-
COPD	12 (14)	51 (15.2)	-	51 (15.2)	18 (5.4)	**	18 (5.4)	12 (14)	-	18 (5.4)	4 (12.5)	-
Psychiatric disorders	1 (1.2)	54 (16.1)	**	54 (16.1)	17 (5.2)	**	17 (5.2)	1 (1.2)	-	17 (5.2)	1 (3.1)	-
Nº medications. Mean (SD)	4.1 (3.6)	3.5 (2.7)	-	3.5 (2.7)	2.5 (2.8)	-	2.5 (2.8)	4.1 (3.6)	**	2.5 (2.8)	5.1 (4.8)	*
Polypharmacy^†^. N (%)	30 (34.9)	106 (31.3)	-	106 (31.3)	60 (18.2)	**	60 (18.2)	30 (34.2)	*	60 (18.2)	11 (34.4)	-
Specific medications. %												
Neuroleptics	3 (3.5)	58 (17.1)	**	58 (17.1)	25 (7.5)	**	25 (7.5)	3 (3.5)	-	25 (7.5)	4 (12.5)	-
Benzodiazepines	12 (14)	96 (28.2)	*	96 (28.2)	51 (15.3)	**	51 (15.3)	12 (14)	-	51 (15.3)	6 (18.8)	-
Hypnotics	2 (2.3)	31 (9.1)	-	31 (9.1)	9 (2.7)	**	9 (2.7)	2 (2.3)	-	9 (2.7)	1 (3.1)	-
Geriatric Syndromes. N (%) SPPB <10	29 (33.7)	62 (18.2)	*	62 (18.2)	32 (9.7)	*	32 (9.7)	29 (33.7)	**	32 (9.7)	9 (28.1)	*
Gait speed												
<0.8m/s	17 (19.8)	17 (5)	**	17 (5)	22 (6.6)	-	22 (6.6)	17 (19.8)	**	22 (6.6)	5 (15.6)	-
≥ 1.2m/s	17 (19.8)	133 (39.1)	**	133 (39.1)	162 (48.9)	-	162 (48.9)	17 (19.8)	**	162 (48.9)	5 (15.6)	**
MOCA ≤ 20	21 (24.4)	29 (8.5)	**	29 (8.5)	38 (11.5)	-	38 (11.5)	21 (24.4)	*	38 (11.5)	5 (15.6)	-
GDS-SF ≥ 6	12 (14)	114 (33.5)	**	114 (33.5)	80 (24.3)	-	80 (24.3)	12 (14)	-	80 (24.3)	4 (12.5)	*
QOL. N (%)												
Fair or poor	45 (52.3)	221 (65)	-	221 (65)	173 (52)	**	173 (52)	45 (52.3)	-	173 (52)	15 (46.9)	-
Not satisfied with his/her life	9 (13.8)	77 (31.6)	*	77 (31.6)	51 (20.6)	*	51 (20.6)	9 (13.8)	-	51 (20.6)	6 (26.1)	-

Comparison of the four resulting groups from the combination of the chronological age and the year of HIV diagnosis with one another. The four groups are: ≤ 1996 < 65; > 1996 < 65; ≤ 1996 ≥ 65 and > 1996 ≥ 65. This table shows the results of four of the 6 resulting comparisons. The *P* set by Bonferroni was .05/6, as we made six comparisons, so *P* values < .0083 were considered statistically significant in this analysis.

*p < .008.

**p < .0001.

–p ≥ 0.0083.

≦1996: HIV diagnosis in 1996 or before. >1996: HIV diagnosis after 1996. ≥65: 65 or over years at the time of recruitment. <65: younger than 65 years old at the time of recruitment. IDU: injection drug user. MSM: men who have sex with men. CDC: Centers for Control Diseases.

*Depression: recorded in the clinical history as a co-morbidity.

^†^ Polypharmacy: ≥ 5 co-medications other than ART.

SPPB: Short Physical Performance Battery. SPPB <10: some functional impairment. MOCA: Montreal Cognitive Assessment test. MOCA ≤20: cognitive impairment. GDS-SF: Geriatric Depression Scale Short Form. GDS-SF ≥ 6: depressive symptoms. QOL: quality of life.

## Discussion

Our study demonstrates important differences among older adults with HIV according to their chronological age and the year of HIV diagnosis concerning comorbidities, frailty, physical function, and QOL.

LTHS comprised almost half of the total patients in our study, but the proportion of patients 65 or over within the HAART-era group was twice that of the LTHS group. This is interesting because HIV care providers, when referring to the older adults with HIV, commonly assume this group is mostly formed by those diagnosed before 1996. We found that LTHS have been living with a known HIV infection for twice as many years; therefore, they were exposed to the toxicity of certain medications until the new era of ART was available. It is worth highlighting there were no differences between them regarding HIV-related factors, but important differences were found regarding comorbidity [19]. We found higher rates of not only comorbidities among LTHS, but also particular diseases: COPD, history of cancer, osteoarthritis, depression, and other psychiatric disorders. COPD is strongly linked to smoking and more prevalent among people with HIV than in general population [20]; it consumes a great deal of resources, and has been demonstrated to negatively impact the QOL of people with HIV [21]. COPD was twice as prevalent within LTHS as in the HAART-era group, and LTHS were more likely to be active smokers. Having a history of cancer was significantly more common among LTHS; in fact, one in ten LTHS in our study were not only HIV survivors, but also cancer survivors. Survival involves experiencing a life-altering event possibly leading to death, isolation, need of support, and the need of reprioritize one’s life [22]. Depression is the most common psychiatric disorder among people with HIV [23] and is a well-known independent factor associated with frailty among older adults with HIV [24], as well as with worse QOL [25]. In an Australian study of 88 LTHS patients analyzed, researchers found depression to be the most common comorbidity in 45% of the patients and other psychiatric disorders were found in 25% of the patients [26]. We found that the prevalence of depression was twice as high in LTHS as in the HAART-era group and the prevalence of other psychiatric illnesses three times as high. According to these data, LTHS took more psychotropic drugs. Interestingly, in our study, benzodiazepines, neuroleptics, and hypnotics use decreased with increasing chronological age, but was twice as high among LTHS as in the HAART-era group. Depression diagnosis is included in the AIDS Survivor Syndrome description, which describes the psychological impact of living with HIV in the first years of the HIV pandemic [27, 28]. In the Global Burden of Disease Study 2019 [29], COPD was the fourth most common cause of burden as measured by the disability-adjusted life-years (DALYs) in the general population aged 50–74 years, just after ischemic heart disease, stroke, and diabetes; depressive disorders and osteoarthritis were among the top 18.

Despite the increased burden of comorbidity, there were no differences between LTHS and the HAART-era group patients regarding frailty and physical function. This reveals that frailty and comorbidity are frequent but not necessarily connected [10]. The main characteristic individuals diagnosed with HIV before 1996 share is that they have survived HIV infection despite the lack of effective treatments in the early years of the pandemic while their peers died [30]. A combination of several host and immune factors must be the reason for their survival, and probably for their reduced risk for frailty. In 1995, before HAART appeared, two studies were published demonstrating that LTHS had low levels of HIV-1 in the presence of strong virus-specific immune responses [31] and that those LTHS with high levels of CD4+ cells maintained control of viral replication but lacked the CD8+ cell activities [32]. The immune system’s role is one of the stress-response systems that regulates homeostasis [12], and inflammation is consistently associated with frailty [33]. Frailty is defined as a state of decreased reserves resulting in increased vulnerability to adverse outcomes when exposed to stressors. It is more common among those with low CD4 counts or a high HIV viral load [34] and is a risk factor for overall mortality [35]. People surviving HIV would have specific characteristics that made them robust and able to maintain homeostasis within the highly and severe stressful situation of an HIV infection without effective treatment.

The impact of pain on QOL is well known [36, 37], and we found it was more prevalent among LTHS. Surprisingly, painkiller prescriptions were not found to be more common among those suffering more pain. This fact underscores that even when pain is assessed, in many cases it is not properly treated. As we have noted before, the comorbidities found more prevalent among LTHS largely affected the patients’ QOL but are not the top comorbidities HIV care providers focus on. Much work remains to be done to seriously face the health-related QOL [38].

On the other hand, HAART-era group individuals, who are in their fifties, were never exposed to the ART’s high toxicity, or the social circumstances of the early years of the AIDS pandemic. Patients 65 or over were 15% of our sample and 75% of them were in the HAART-era group. They showed no differences from the chronologically younger patients regarding virological response to ART, but did have a significantly lower CD4+ T-cell nadir count were found, which has largely been described among older adults due to the delay in the diagnosis [39] and lower current CD4+ T-cell count. This is consistent with evidence establishing a lower and slower immunological response to ART increasing with chronological age [40]. This mitigated immune response seems to have more to do with age-related aging [41] than with HIV-related immunosenescence since the years with known HIV infection were significantly lower among patients 65 or over.

HAART-era indivuals’ profiles regarding comorbidity were radically different from those of LTHS. All the comorbidities that were more prevalent in those 65 or over, except for osteoarthritis, were registered in the AIDS Therapy Evaluation in the Netherlands (ATHENA) cohort study and showed an exponential increase with age among adults with HIV in the predictive model [6]. Multimorbidity increases with age [42], but as mentioned before, it is particularly important to consider the heterogeneity of chronic diseases implicated in multimorbidity to describe their specific burden of disease and impact on QOL because the number of comorbidities alone appears not to add qualitative value [43].

Frailty was twice as high in patients 65 or over than in the younger patients, which is consistent with the nature of frailty phenotype that is intrinsically related to aging and whose pathophysiological substrate is linked to inflammageing [12]. The same occurs with physical impairment, as measured by SPPB and gait speed. Nevertheless, it is worth highlighting that one in five of the patients 65 or over had a gait speed greater than 1.2m/s, which reflects a good health and functional status and suggests better-than-average life expectancy [9]. This fact supports the main objective of this study, which was intended to demonstrate that older adults with HIV are not a homogeneous group, and it is necessary to stratify them with objective measures of physical function and frailty.

Our study’s limitations include the nature of observational research; it cannot establish a relationship of causality between variables. The main strength is the number of older adults with HIV included in the multicenter cohort study and the exhaustive assessment performed on all of them, covering not only comorbidity, but also frailty, physical function, other geriatric syndromes, and QOL. Older adults with HIV are currently the largest population group among people with HIV, even though most studies thus far have focused on them as a homogeneous group, which might prompt misleading conclusions.

## Conclusions

An LTHS and a person 65 or older are both “older adults with HIV,” but their characteristics and requirements differ markedly. The data our study provided define specific profiles of patients among older adults with HIV to aid clinicians, researchers, and health system managers to further design specific approaches focused on their real needs.

## Supporting information

S1 Table Comparison by groups combining chronological age and year of HIV diagnosis: Patients 65 or over diagnosed pre vs post-1996 and, patients diagnosed pre-1996 <65 vs 65 or over. (DOCX)

S2 Table Data supporting the information shown in Figs 1 and 2: Comorbidities, frailty, physical function, other geriatric syndromes, and quality of life stratified both by chronological age and year of HIV diagnosis. (DOCX)
