# Supplementary material for: Different profiles among older adults with HIV according to their chronological age and the year of HIV diagnosis: The FUNCFRAIL cohort study (GeSIDA 9817)
Source: PLoS One. 2022 Mar 30;17(3):e0266191. doi: 10.1371/journal.pone.0266191 (PMC8967027; doi:10.1371/journal.pone.0266191)
Supplement: S1 Table — (DOCX) [file pone.0266191.s001.docx]

**S1 Table. Comparison by groups combining chronological age and year of HIV diagnosis: patients 65 or over diagnosed pre vs post-1996 and, patients diagnosed pre-1996 < 65 vs 65 or over.**

|  | > 65 pre-1996 vs post-1996 | | | Pre-1996 < 65 vs > 65 | | | |  | |
| --- | --- | --- | --- | --- | --- | --- | --- | --- | --- |
|  | ≤ 1996 > 65 | > 1996 > 65 | p | ≤ 1996 < 65 | ≤ 1996 > 65 |  | |  | |
| Patients. N | 32 | 86 |  | 340 | 32 |  | |  | |
| Women. N (%) | 5 (15.6) | 19 (22.1) | - | 110 (32.4) | 5 (15.6) | - | |  | |
| Living alone. N (%) | 18 (56.3) | 40 (46.5) | - | 96 (28.3) | 18 (56.3) | * | |  | |
| Age at HIV diagnosis.  Median (p25-75) | 43.5  (39.6-46.3) | 56.6  (52-61.5) | ** | 27.5  (24.4-31) | 43.5  (39.6-46.3) | ** | |  | |
| Years with known HIV infection. Median (p25-75)) | 26.1  (23.9-28.3) | 14.2  (9.8-18.3) | ** | 28.3  (25.3-31.1) | 26.1  (23.9-28.3) | * | |  | |
| Risk practice for HIV infection. N (%)  IDU  MSM  Heterosexual | 4 (12.5)  17 (53.1)  8 (25) | 2 (2.3)  40 (46.5)  31 (36) | -  -  - | 221 (65.2)  43(12.7)  58 (17.1) | 4 (12.5)  17 (53.1)  8 (25) | **  **  - | |  | |
| B CDC stage. N (%) | 2 (6.9) | 9 (10.8) | - | 82 (24.6) | 2 (6.9) | - | |  | |
| Nadir CD4+ T-cell. Median (p25-75) | 197.5  (119.5-324) | 160  (71-260) | - | 180  (87-287) | 197.5  (119.5-324) | - | |  | |
| Current CD4+ T-cell. Median (p25-75) | 721.4  (517.3-947) | 571  (441-748) | - | 685  (487-946) | 721.4  (517.3-947) | - | |  | |
| Current smoker. N (%) | 7 (22.6) | 12 (14.1) | - | 188 (56.5) | 7 (22.6) | ** |  | |  |
| Nª comorbidities. Mean (SD)  Comorbidities. N (%)  < 2  3-4  > 5 | 2.8 (1.8)  17 (53.1)  12 (37.5)  3 (9.4) | 2.5 (1.6)  47 (54.7)  28 (32.6)  11 (12.8) | -  -  -  - | 2.6 (1.8)  176 (51.8)  109 (32.1)  55 (16.2) | 2.8 (1.8)  17 (53.1)  12 (37.5)  3 (9.4) | -  -  -  - |  | |  |
| Specific comorbidities. %  Hypertension  Diabetes Mellitus  Cancer  Osteoarthritis  Depression*  COPD  Psychiatric disorders | 17 (53.1)  9 (28.1)  2 (6.3)  9 (28.1)  1 (3.1)  4 (12.5)  1 (3.1) | 33 (38.4)  20 (23.3)  15 (17.6)  26 (30.2)  9 (10.6)  12 (14)  1 (1.2) | -  -  -  -  -  -  - | 100 (29.6)  50 (14.8)  21 (6.2)  83 (25)  75 (22.3)  51 (15.2)  54 (16.1) | 17 (53.1)  9 (28.1)  2 (6.3)  9 (28.1)  1 (3.1)  4 (12.5)  1 (3.1) | -  -  -  -  *  -  - |  | |  |
| Nº medications. Mean (SD)  Polypharmacy^†^. N (%)  Specific medications. %  Neuroleptics  Benzodiazepines  Hypnotics | 5.1 (4.8)  11 (34.4)  4 (12.5)  6 (18.8)  1 (3.1) | 4.1 (3.6)  30 (34.9)  3 (3.5)  12 (14)  2 (2.3) | -  -  -  -  - | 3.5 (2.7)  106 (31.3)  58 (17.1)  96 (28.2)  31 (9.1) | 5.1 (4.8)  11 (34.4)  4 (12.5)  6 (18.8)  1 (3.1) | -  -  -  -  - |  | |  |
| Geriatric Syndromes. N (%)  SPPB <10  Gait speed  <0.8m/s  ≥ 1.2m/s  MOCA < 20  GDS-SF > 6 | 9 (28.1)  5 (15.6)  5 (15.6)  5 (15.6)  4 (12.5) | 29 (33.7)  17 (19.8)  17 (19.8)  21 (24.4)  12 (14) | -  -  -  -  - | 62 (18.2)  17 (5)  133 (39.1)  29 (8.5)  114 (33.5) | 9 (28.1)  5 (15.6)  5 (15.6)  5 (15.6)  4 (12.5) | -  -  *  -  * |  | |  |
| QOL. N (%)  Fair or poor  Not satisfied with his/her life | 15 (46.9)  6 (26.1) | 45 (52.3)  9 (13.8) | -  - | 221 (65)  77 (31.6) | 15 (46.9)  6 (26.1) | -  - |  | |  |

The *P* set by Bonferroni was .05/6, as we made six comparisons, so *P* values < .0083 were considered statistically significant in this analysis. *p < .008. **p < .0001. – p > 0.0083. ≤1996: HIV diagnosis in 1996 or before. >1996: HIV diagnosis after 1996. >65: 65 or over years at the time of recruitment. <65: younger than 65 years old at the time of recruitment. IDU: injection drug user. MSM: men who have sex with men. CDC: Centers for Control Diseases. *Depression: recorded in the clinical history as a co-morbidity. ^†^ Polypharmacy: ≥ 5 co-medications other than ART. SPPB: Short Physical Performance Battery. SPPB <10: some functional impairment. MOCA: Montreal Cognitive Assessment test. MOCA <20: cognitive impairment. GDS-SF: Geriatric Depression Scale Short Form. GDS-SF > 6: depressive symptoms. QOL: quality of life
