# Supplementary material for: Different profiles among older adults with HIV according to their chronological age and the year of HIV diagnosis: The FUNCFRAIL cohort study (GeSIDA 9817)
Source: PLoS One. 2022 Mar 30;17(3):e0266191. doi: 10.1371/journal.pone.0266191 (PMC8967027; doi:10.1371/journal.pone.0266191)
Supplement: S2 Table — (DOCX) [file pone.0266191.s002.docx]

**S2 Table. Data supporting the information shown in Fig 1 and 2: comorbidities, frailty, physical function, other geriatric syndromes, and quality of life stratified both by chronological age and year of HIV diagnosis.**

|  | Total | Chronological Age | | |  | Year of HIV diagnosis | |  |
| --- | --- | --- | --- | --- | --- | --- | --- | --- |
|  |  | 50-54 | 55-64 | > 65 | p | < 1996 | >1996 | p |
| Patients. N (%) | 801 | 290 (36.2) | 393 (49) | 118 (14.7) |  | 372 (47.0) | 419 (52.9) |  |
| Comorbidities. N (%)  Hypertension  Diabetes Mellitus  Dyslipidemia  Osteoarthritis  Chronic kidney disease  Cancer  Depression*  Psychiatric disorders  COPD  History of cancer | 230 (28.8)  108 (13.6)  349 (43.8)  167 (21.1)  52 (6.5)  55 (6.9)  124 (15.6)  73 (9.2)  87 (10.9)  59 (7.4) | 70 (24.4)  25 (8.7)  102 (35.5)  50 (17.7)  18 (6.3)  24 (8.4)  42 (14.7)  30 (10.5)  28 (9.8)  15 (5.2) | 110 (28.1)  54 (13.8)  187 (47.8)  82 (21.2)  19 (4.9)  14 (3.6)  72 (18.4)  41 (10.6)  43 (11.1)  32 (8.2) | 50 (42.2)  29 (24.6)  60 (50.8)  35 (29.7)  15 (12.7)  17 (14.1)  10 (8.5)  2 (1.7)  16 (13.6)  12 (10.2) | 0.001  0.001  0.002  0.02  0.01  0.001  0.03  0.01  0.5  0.1 | 117 (31.6)  59 (16)  165 (44.6)  92 (25.3)  28 (7.6)  23 (6.2)  76 (20.7)  55 (15)  50 (15)  36 (9.7) | 111 (26.6)  48 (11.5)  181 (43.3)  74 (17.8)  24 (5.8)  32 (7.7)  47 (11.3)  18 (4.4)  30 (7.2)  23 (5.5) | 0.1  0.07  0.7  0.01  0.3  0.4  0.001  0.001  0.001  0.02 |
| Geriatric Syndromes  Frailty  Gait speed < 0.8m/s  SPPB < 10  MOCA < 20  GDS-SF > 6 | 46 (5.7)  65 (8.1)  133 (16.7)  96 (12.8)  213 (26.5) | 13 (4.5)  18 (6.3)  36 (12.5)  26 (9)  80 (27.9) | 20 (5.1)  25. (6.4)  59 (15.1)  44 (11.2)  117 (29.9) | 13 (11)  22 (18.6)  38 (32.2)  26 (22)  16 (13.5) | 0.02  0.001  0.001  0.001  0.007 | 18 (4.8)  22 (5.9)  71 (19.1)  34 (9.1)  118 (31.7) | 27 (6.5)  39 (9.4)  61 (14.7)  59 (14.1)  92 (22.1) | 0.3  0.08  0.1  0.03  0.001 |
| Quality of life  Fair or poor  Not satisfied with his/her life  Pain | 461 (57.5)  144 (17.9)  164 (20.4) | 173 (59.7)  58 (28.4)  65 (38.5) | 228 (58)  71 (24.1)  78 (35.5) | 60 (50.8)  15 (17)  21 (29.6) | 0.2  0.1  0.4 | 236 (63.4)  83 (31.7)  82 (41.2) | 218 (52)  60 (19.2)  81 (31.5) | 0.001  0.001  0.03 |

*Depression: recorded in the clinical history as a co-morbidity. SPPB <10: some functional impairment. MOCA: Montreal Cognitive Assessment test. MOCA < 20: cognitive impairment. GDS-SF: Geriatric Depression Scale Short Form. GDS-SF > 6: depressive symptoms.
